# Comparison of hyperfibrinolysis in trauma and non-trauma patients

**DOI:** 10.1186/2197-425X-3-S1-A851

**Published:** 2015-10-01

**Authors:** T Madelaine, G Marcotte, L Rugeri, J-X Taverna, D Massignon, B Floccard, T Rimmelé, J-S David

**Affiliations:** Hospices Civils de Lyon, Department of Anesthesiology and Intensive Care, Groupement Hospitalier Edouard Herriot, Lyon, France; Hospices Civils de Lyon, Department of Hemostasis, Hôpital Louis Pradel, Lyon, France; Hospices Civils de Lyon, Department of Anesthesiology and Intensive Care, Groupement Hospitalier Lyon-Sud, Lyon, France; Hospices Civils de Lyon, Department of Hemostasis, Groupement Hospitalier Lyon-Sud, Lyon, France

## Introduction

Hyperfibrinolysis (HF) is a major issue of trauma-induced coagulopathy but has also been described in many other medical or surgical settings such as cardiopulmonary surgery, gastro-intestinal bleeding, liver transplantation and post-partum hemorrhage.

## Objectives

The aim of this study was to describe the HF in a cohort of patients experiencing severe bleeding from trauma and non-trauma source and to highlight the differences between both groups.

## Methods

We conducted an observational, retrospective study in two academic trauma centers from a French university institution. All patients diagnosed with HF on rotational thromboelastometry (ROTEM^®^) between 2010 and 2014 were included. Demographic and clinical parameters, standard laboratory and ROTEM^®^ results were collected. HF diagnosis was established when the 60 minute clot lysis index (CLI_60_) was below 85% of the maximum clot firmness. HF was classified as fulminant HF (immediate breakdown of the clot within 30 minutes), intermediate HF (breakdown of the clot between 30 and 60 minutes) and late HF (complete clot lysis after more than 60 minutes). Data are expressed as n (%) and median [interquartile range].

## Results

**Figure 1:**
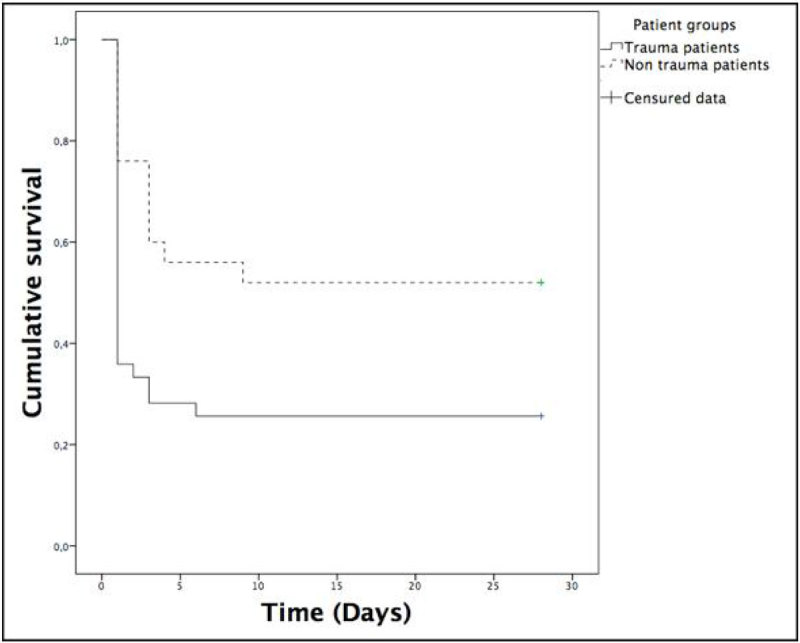
**Twenty-eight day survival of trauma and non trauma**.

Sixty-four patients were included (39 trauma and 25 non trauma). Overall mortality was 64% (95% confidence interval [CI], 52 to 76). Median ISS for trauma patients was 49 (95% CI, 42 to 55). Patients severity assessed with SAPS II was not different between groups (median [IQR] for trauma 76 [23] vs. non trauma 57 [47]; p = 0.36) whereas 28 day survival was greater in non-trauma patients (53% [95% CI, 31 to 73] vs. 26% [95% CI, 11 to 40]; p = 0.03) (Figure 1). More fulminant HF were observed in the trauma group (p < 0.01). Survival was also different according to the type of HF and was inferior in the fulminant group (14% [95% CI, 0 to 27] vs. intermediate: 25% [95% CI, 0 to 54] vs. late: 70% [95% CI, 49 to 90]; p < 0.001). During the study period, HF incidence in trauma patients decreased from 2.9 for 100 patients-years (2011) to 1.6 (2012) and 0.4 (2013) with no significant variation in trauma severity (ISS: 21 [95% CI, 20 to 23] (2011), 19 [95% CI, 18 to 20] (2012) and 20 [95% CI, 19 to 21] (2013); p = 0.30).

## Conclusions

Fulminant HF is more frequent in trauma patients and is associated with an increased mortality. The decreasing incidence of HF observed during the study period might be due to the systematic prehospital administration of tranexamic acid in trauma patients since 2011 in our city area.

